# Ribosomal Protein L10: From Function to Dysfunction

**DOI:** 10.3390/cells9112503

**Published:** 2020-11-19

**Authors:** Daniela Pollutri, Marianna Penzo

**Affiliations:** 1 Department of Experimental, Diagnostic and Specialty Medicine Alma Mater Studiorum University of Bologna, Via Massarenti 9, 40138 Bologna, Italy; daniela.pollutri2@unibo.it; 2 Center for Applied Biomedical Research (CRBA), Alma Mater Studiorum University of Bologna, Via Massarenti 9, 40138 Bologna, Italy

**Keywords:** RPL10, ribosome, cancer, ribosomopathy, rare disease, translation, protein synthesis

## Abstract

Eukaryotic cytoplasmic ribosomes are highly structured macromolecular complexes made up of four different ribosomal RNAs (rRNAs) and 80 ribosomal proteins (RPs), which play a central role in the decoding of genetic code for the synthesis of new proteins. Over the past 25 years, studies on yeast and human models have made it possible to identify *RPL10* (ribosomal protein L10 gene), which is a constituent of the large subunit of the ribosome, as an important player in the final stages of ribosome biogenesis and in ribosome function. Here, we reviewed the literature to give an overview of the role of RPL10 in physiologic and pathologic processes, including inherited disease and cancer.

## 1. Introduction

Eukaryotic cytoplasmic ribosomes are highly structured macromolecular complexes made up of four different ribosomal RNAs (rRNAs) and 80 ribosomal proteins (RPs), which play a central role in the decoding of genetic code for the synthesis of new proteins. These two distinct yet complementary functions are separated into the two ribosomal subunits: the 40S (or small) and the 60S (or large) subunits, respectively. Even though the catalytic activity of ribosomes is held by rRNAs, many of the RPs are known to be essential for different steps and aspects of translation [1].

The process of ribosome production, *ribosome biogenesis*, is a highly coordinated process that takes place in the nucleolus, nucleoplasm, and cytoplasm. It involves (I) the transcription of rRNAs, which are extensively modified by a number of enzymatic complexes and enzymes, (II) the synthesis of RPs, and (III) the step-by-step assembly of rRNAs and RPs to form the mature large and small subunits (reviewed in [1]). Ribosome biogenesis is a highly energy-consuming process. Therefore, it is of great importance for the cell to synthesize all the components in a stoichiometric proportion; this goal is achieved primarily by means of a coordinated expression of the ribosomal proteins [2,3], which then participate in the regulation of rRNA transcription and modification [2,4]. Furthermore, to produce a sufficient number of functionally competent ribosomes, the cell has developed a number of quality checkpoints, which have been only partially identified to date [5]. Nevertheless, the emergence of variants of ribosomal constituents related to acquired or somatic mutations has been documented in several pathological settings [6,7,8]. In parallel, the old vision of “the ribosome” as a univocally determined, passive protein factory, has been replaced by the evidence-based awareness of ribosome diversity as a source of an additional level of gene expression control [6,7]. In this sense, the ribosome compositional diversification may be linked to physiological processes, such as cellular growth and differentiation, as well as to pathological conditions.

Among the ribosome constituents that frequently undergo mutations in disease, there is RPL10 (uL16 in the new nomenclature [9]), also known as QM or DXS648 [10,11,12]. Although *RPL10* mutations have been reported in numerous inherited and acquired diseases, their impact on RPL10 ribosomal and extra-ribosomal functions, and, consequently, their role in the pathogenetic processes, has not been completely determined. Here, we reviewed the literature concerning the known functions and alterations of RPL10, focusing on the studies conducted in the past decades on eukaryotic models.

## 2. RPL10 Gene and Protein

**Figure 1:**
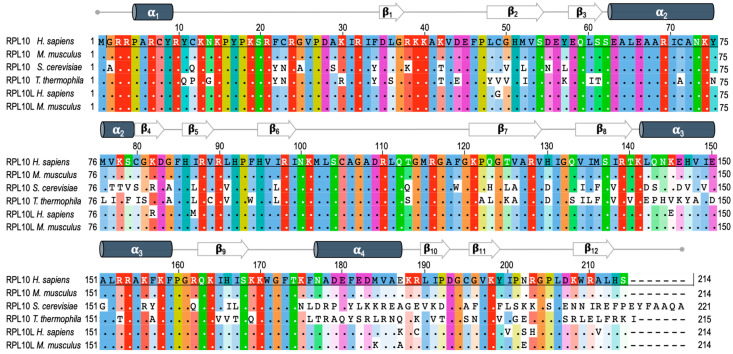
RPL10 and RPL10L (ribosomal protein L10-like gene) protein sequence alignment. The figure shows the multi-sequence alignment of RPL10 protein from *Homo sapiens* (P27635), *Mus Musculus* (Q6ZWV3), *Saccharomyces cerevisiae* (P41805), and *Tetrahymena thermophila* (Q235M8). In addition, the sequences of RPL10L from *Homo sapiens* (Q96L21) and *Mus musculus* (P86048) have been aligned. Sequences were aligned using Jalview program [17]. Each residue box in the alignment is assigned a color according to ClustalX color scheme. The higher the intensity of the color, the higher the conservation of the residue (white = not conserved residue) with respect to human RPL10. At the top of alignments the secondary structure of RPL10 from *Tetrahymena thermophila* is reported, which is visualized using PyMOL tool, as shown in Figure 2.

RPL10 is encoded by the *RPL10* gene located on the X chromosome. RPL10 is listed among the L10e family members, which are the structural and functional orthologs of the L16 family in Eubacteria [13]. In a study on sequence changes in the *RPL10* gene over the course of evolution, Klauck and colleagues found that the translation product of the gene has a highly conserved amino acid sequence from yeast to human (about 67% protein identity, Figure 1) and archaea (about 43% identity) [13]. This is true in spite of remarkable genetic differences, such as variable patterns in both exons and introns length, and in GC content, probably leading to different splicing and epigenetic events [14]. For example, mouse RPL10 differs from its human homolog in only one residue (Figure 1). Such an evolutionary conservation in the aminoacid sequence indicates the essential nature of RPL10 in the biology of the organisms during the evolution. Although an increasing number of RPL10 extra-ribosomal functions are emerging, the strategic position close to catalytic center and regulatory sites within the ribosome evidences a fundamental role of this protein in ribosome integrity and function [13]. Indeed, the essential role of this protein is supported by the identification of an autosomal intron-less *RPL10* homolog named *RPL10L* (ribosomal protein L10-like gene), mapping on chromosome 14 and showing about 96% identity in the aminoacid sequence (Figure 1) and a 90% identity in the coding sequence compared to *RPL10*. The identity level was determined by comparing the cDNA sequences of the two genes. Northern blot analysis and PCR assays conducted by Uechi and colleagues in 16 human tissues and eight tumor-derived cell lines, showed a testis-specific expression of RPL10L. Since this gene has no introns in its coding region, they concluded that it was most likely produced by retro-transposition of the original X-linked gene in the course of the evolution to compensate for the reduced dosage during spermatogenesis and to escape the meiotic sex chromosome inactivation (MSCI) [15]. Indeed, RT-PCR assays of *Rpl10L* in male mouse germ cells at different developmental stages showed an increase in *Rpl10L* expression during meiosis specifically when *Rpl10* is silenced by MSCI. Further experiments conducted by Jiang and colleagues using mouse and human cellular models made it possible to gain further insights into the function of this retroposon. Mouse RPL10L shows about 99% identity in the aminoacid sequence compared to RPL10 and about 95% identity in the aminoacid sequence respect to its human ortholog and is located in the same syntenic region of the genome (Figure 1) [16].

In the Jiang models, *Rpl10L* deficiency affected ribosome biogenesis and blocked spermatogenesis in the late-prophase, resulting in *Rpl10L*^−/−^ male mice infertility, but transgenic *Rpl10* rescue restored spermatogenesis and fertility. On the other hand, ectopically expressed RPL10 or RPL10L prevented the death of RPL10-deficient human cells to the same extent, thus demonstrating that RPL10L and RPL10 are interchangeable in these models [16]. In this context, it is worth pointing out that even though these rescue experiments highlighted a functional identity of the two proteins, these authors did not formally demonstrate whether RPL10L could replace RPL10 within ribosomes. However, in this regard, cryo-electron microscopy (cryo-EM) analyses performed on human ribosomes from HeLa cells highlighted the presence of RPL10L in place of RPL10 [18].

**Figure 2:**
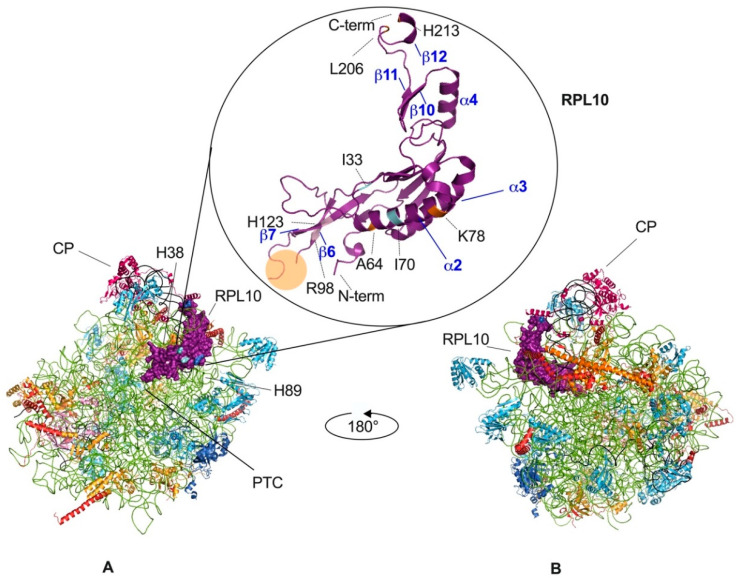
Eukaryotic large ribosomal subunit and RPL10 mutations. *Tetrahymena termophila* 60S subunit, views of the 40S-exposed (**A**) and solvent-exposed (**B**) sides. RPL10 is in purple, 25S rRNA is in green, 5S is rRNA in black, and 5.8S is in carbon (visible in panel B). The localization of the central protuberance (CP), of the peptidyl-transferase center (PTC) and of other elements, which are significant for the present review are pinpointed in the 40S-exposed view. In the circle, the structure of the RPL10 protein is magnified, while clinically significant mutations are highlighted in different colors (pale pink for the mutations involved in T-ALL, light blue for the mutations involved in multiple myeloma, orange for the mutations involved in CNS-related abnormalities). The P-loop (between residues 102 and 112, orange circle) cannot be visualized because no ligand is present in this structure, as also explained in the main text. Some of the α helices and β sheets have been labeled, according to the numbering in Figure 1. The figure was generated using PyMOL (Schrodinger, LLC).

Within the ribosome, RPL10 is located on the inter-subunit side of the large subunit (Figure 2), which is almost completely covered by the small subunit in the 80S ribosome, and it shows topological characteristics largely conserved from yeast to human, which have been described here for yeast. In the *Saccharomyces cerevisiae* model, the small subunit (40S) comprises 33 ribosomal proteins and the 18S ribosomal RNA (rRNA), whereas the large subunit (60S) comprises 46 ribosomal proteins and three rRNAs (25S, 5.8S, and 5S rRNA). Within mature, large ribosomal subunits, RPL10 extends from the central protuberance—where its N-terminus is inserted between helices 89 and 38 (H89, H38) of 25S rRNAs—to the ribosomal stalk, which is a structure formed by a complex of three proteins and 25 rRNA, where the eukaryote-specific C-terminal region of RPL10 extends to the solvent-accessible side near the factor-binding site, providing a binding site for regulatory proteins [19,20,21]. In this region, RPL10 is close to the sarcin-ricin loop and to the GTPase center (GAC) [21,22] that serves as a factor-binding site essential for GTP-catalyzed steps of the initiation and elongation cycles of translation [23]. The RPL10 portion internal to the ribosome is positioned across the corridor, where every incoming tRNA must pass by, and it extends down into the peptidyl-transferase center (PTC), which is the active site of the large subunit, where the formation of peptide bonds and the hydrolysis of peptidyl-tRNA bonds occur. In this region, RPL10 inserts with a conserved internal loop also referred to as the P-site loop, which is hardly visible, if at all, in cryo-EM or crystallographic structures, indicating that it is disordered and is consequently not involved in stable contacts within the large subunit (Figure 2) [19,22,24]. Nevertheless, the P-site loop becomes ordered upon the binding of specific ligands such as t-RNAs [25] or assembly factors such as SBDS (Shwachman-Bodian-Diamond Syndrome) [26]. This portion of the protein, consisting of amino acids from 102 to 112, plays a role in the ribosome conformational shift between the non-rotated and the rotated state, which promptly occurs during the elongation cycle of translation [21,24].

Taken together, the high conservation throughout evolution, the unique structural properties, and the fine-tuned connections with all the functional and regulatory centers of the ribosome gave rise to the interest in eukaryotic RPL10 as a pivot of translational control of gene expression [27,28].

## 3. RPL10 Ribosomal Functions

As mentioned earlier, RPL10 is a structural constituent of the large subunit of the ribosome (Figure 2). A series of studies investigating mutant variants of RPL10 have shown that from yeast to human, RPL10 is a functionally conserved ribosomal protein, which performs multiple ribosome-associated functions [19,24,29]. Nevertheless, several extra-ribosomal functions have also been reported for RPL10, which will be discussed below.

Thanks to the high level of *RPL10* conservation throughout evolution, studies of yeast models have made it possible to gain most of the knowledge we have on the physiological ribosome-associated functions of RPL10. To date, it has been established that RPL10 is required for ribosome biogenesis, being essential for 60S subunit biogenesis, nuclear export (albeit indirectly), and joining with the 40S subunit, while it is actively involved in the rotation of subunits during the elongation, in aminoacyl tRNA movement, and in translation control [24,30].

Ribosome biogenesis is a complex process consisting of a hierarchical sequence of steps, proceeding through the formation of a series of intermediates that mature step by step, and culminating in the production of functionally active ribosomes. The pre-ribosomal particles assembly starts in the nucleolus, where rRNAs are transcribed, processed, and begin to be assembled to ribosomal proteins and trans-acting biogenesis factors, which guide the subsequent maturation process. Then, immature ribosomal subunits are exported from the nucleolus into the nucleus where pre-rRNA processing is completed, and a pre-60S ribosome containing the mature 25S, 5.8S, and 5S rRNAs is exported to the cytoplasm.

The final maturation of the 60S subunit in the cytoplasm involves the release and recycling of several trans-acting factors and the incorporation of the remaining ribosomal proteins, so that mature 60S and 40S can finally join to form functionally active ribosomes [24,31,32,33]. The incorporation of RPL10 in the large subunit is a late step in this maturation process and takes place in the cytoplasm. Recently, high-resolution structures of pre-60S intermediates at different assembly stages have been reported [34,35,36]. These intermediate structures, that represent the assembly steps immediately before and after nuclear export, helped to define the dynamic changes leading to and resulting from RPL10 incorporation [34,35,36]. In the nucleus, immature large subunits lacking Rpl10 are complexed with the export adapter protein Nmd3 that recruits the nuclear export receptor Xpo1 (Crm1 in human) through a C-terminal nuclear export signal sequence and exports the pre-60S in the cytoplasm [34]. Cryo-EM analyses of Nmd3-containing pre-60S particles showed an “early-cytoplasmatic intermediate” in which Nmd3 is positioned in the inter-subunit face of 60S, spanning from the L1 stalk, through the tRNA corridor, at the sarcin-ricin loop [34,35]. In this stage, Nmd3 occupies the A, P, and E sites of the tRNA channel, where it would prevent an association of ligands for these sites [37] and contact Tif6 (eIF6 in human), which is an assembly factor that sterically blocks the association of the 40S [38,39,40]. Therefore, its removal is essential for the assembly of translationally active 80S ribosomes in the cytoplasm [29,41]. Moreover, Nmd3 interacts and captures H38 and H89, which are the two helices that form the binding cleft for RPL10 [35]. Thus, Nmd3 seems to be involved in the interactions with different important functional regions to prevent immature subunits from engaging prematurely with the translational machinery.

The subsequent binding of the GTPase Lsg1 to the N-terminal domain of Nmd3 releases H89, thus allowing the insertion of Rpl10, which in turn promotes the rotation of H38 away from Nmd3. The release of H38 further stabilizes Rpl10 in its binding cleft. The P-site loop of Rpl10 can now fully accommodate in the PTC, competing with Nmd3 for an overlapping binding site in the P-site and pushing for Nmd3 dissociation [34]. RPL10 incorporation into the PTC (Figure 2) promotes binding of the tRNA structural mimic Sdo1 (SBDS in human), which is required for the release of Tif6 through recruitment of the GTPase Efl1 (EFL1 in human, a paralog of eukaryotic elongation factor 2—eEF2) [26]. Sdo1 and Efl1 binding represents a quality check to ensure that only properly functioning 60S subunits are licensed for translation. Mutations in the SBDS gene are at the basis of the ribosomopathy Shwachman-Diamond Syndrome (SDS, see below) [42]. Upon eIF6/Tif6 release, the two subunits joining is allowed [43]. RPL10 mutation and depletion studies demonstrated that large subunits lacking RPL10 are functionally inactive and unable to form 80S ribosomes [22,24,37,44,45].

**Figure 3:**
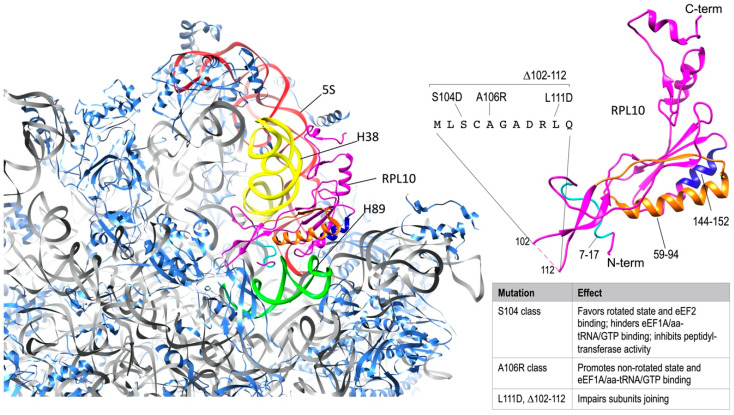
RPL10 ribosomal functions. On the left, Rpl10 in *S. cervisiae* 60S subunit (40S-exposed side); 25S rRNA is represented in grey (except for the colored portions, explained below) and RPLs other than Rpl10 are represented in cornflower blue. The regions of Rpl10 (magenta) that are relevant for interactions with rRNAs have been highlighted with different colors as follows: the portion including aminoacids 7-17 (light blue) is involved in contacting H89 in 25S rRNA (green); the portion including aminoacids 59-94 (orange) is placed between H38 in 25S rRNA (yellow) and H39 in 18S rRNA (not shown); the portion including aminoacids 144-152 (blue) is in close proximity of H89 (green); the C-terminal region is in contact with 5S rRNA (red). On the right side of the figure, Rpl10 structure is magnified and consistently colored. The unresolved sequence of the P-loop, with the different classes of mutations, as reported in the main text, has been added and the effects of the mutations in this site have been summarized in the side table. The figure was generated using UCSF Chimera [46].

Apart from taking part in the arrangement of the PTC (Figure 2), RPL10 also acts as a sensor of activity, transducing information to other functional centers to coordinate ribosome function [19,24,35]. Another extensive mutational analysis of RPL10 from yeast found that Rpl10 proteins with mutations in the P-site loop were unable to bind to the ribosome and accumulated in the nucleus [22]. As a consequence, the release of Nmd3 was impaired. Meanwhile, Rpl10 proteins completely lacking the central loop (Rpl10 Δ102–112) and a specific point mutant (Rpl10 L111S) were incorporated into 60S, but those subunits were not observed in 80S ribosomes or polysomes [22] due to the impaired release of eIf6, which in turn inhibits subunits joining. To define the implications of different regions of RPL10 on ribosome functional activity, Petrov and colleagues generated a set of *rpl10* mutants in a strain of *Saccharomyces cerevisiae*, which is highly sensitive to translational apparatus defects [44]. In this study, areas of Rpl10 with high frequencies of mutations were clustered in three regions of the protein sequence, each one covering the amino acids 7–17, 59–94, and 144–152, respectively, and divided into four spatially defined groups in the folded protein (Figure 3). In group one, mutations predominantly struck residues in a hook-like structure located at the N-terminal region of Rpl10, which comes into contact with the PTC-proximal bulge of 25S rRNA helix H89. The second group was placed between H38 and H39 in 25S and 18S rRNA, respectively, in close proximity to the so called “A-site finger” in H38 [22]. Group three was in a region of the protein lying over helix 89. Lastly, group four was located in the structurally unresolved C-terminal region of Rpl10, in contact with 5S rRNA, which in turn interacts with Rpl5 and Rpl11 at the top of the central protuberance and extends toward the solvent-accessible side, near the initiation and elongation factor-binding sites [24,44] (Figure 3). Consistent with other findings, no mutants mapping to the unstructured central loop (amino acids 102–112) were identified, which was most likely because this loop, which closely approaches the PTC, is so important for the peptidyl-transferase activity that mutants in this region are inviable [22].

A detailed analysis of the mutants located in the N-terminal region of Rpl10 revealed strong effects on the rRNA structure impairing tRNA movement through the large subunit during the elongation cycle [44], thus suggesting a role played by this region in the cyclic conformational changes occurring during the elongation.

In a further mutagenesis study within the internal loop region of the yeast *rpl10* gene, Bussiere and colleagues generated a series of mutants [45] that were subsequently grouped into two major classes [24], both impairing the prompt rotational movement underlying the two conformational states of the ribosome, but with opposite effects. The first class, called the S104D-like class (related to the representative mutant S104D) failed to release the anti-association factor Tif6 with a consequent 60S maturation defect and improper subunit joining (Figure 3). Ribosomes of this class were prone to assume the rotated state, favoring the binding of eEf2 (elongation factor 2), destabilizing binding of eEf1A/aa-tRNA/GTP (elongation ternary complex), and inhibiting peptidyl-transferase activity. The second class, called the A106R-like class, promoted the non-rotated state, facilitating elongation ternary complex binding, stimulating the binding of tRNAs to the A-site, and leaving peptidyl-transferase activity unaffected (Figure 3). The disequilibrium between the rotational conformational states of ribosomes ultimately impacted on different aspects of translation. These were the maintenance of translational reading frame, of translational fidelity (the ability to discriminate between cognate, near-cognate, and non-cognate codons), and the termination codon recognition [24].

During the elongation phase, RPL10 coordinates allosteric movements that occur in the core of 60S, underlying the transition of ribosomes between non-rotated and rotated state, and the tRNAs movement. Indeed, mutational studies in yeast, discussed above, identified Rpl10 mutants impacting the natural equilibrium between the two conformational states [24] and the movement of tRNA from the P-site to the E-site and the release of deacylated tRNA from the ribosome [44], indicating that RPL10 is a central controller of these processes.

In summary, a number of studies conducted in yeast models have shown that RPL10 is essential for achieving complete 60S subunit maturation and subunit joining, as well as for preserving several aspects of ribosomes catalytic activity. Therefore, it is not surprising, as discussed later, that alterations in RPL10 are involved in pathological processes.

## 4. RPL10 Extra-Ribosomal Functions

As for many other ribosomal proteins, there is growing evidence that RPL10 has other functions apart from ribosome biogenesis and protein synthesis, even though in the case of RPL10, most of them are circumstantial and not yet well defined, and they could possibly be consequences of RPL10’s ribosomal functions. The observation that RPL10 is more highly expressed in embryonic rapidly dividing tissues than in adult differentiated ones first suggested the involvement of RPL10 in development and differentiation [12,47]. Moreover, a growing number of studies reported a direct involvement of RPL10 in signal transduction pathways with a role in cell biological processes, such as cell proliferation, migration, differentiation, apoptosis, and metabolism regulation [10,12,48,49,50]. Human RPL10 and its chicken homolog (previously named Jif-1, Jun interacting factor 1) negatively regulate c-Jun by inhibiting its DNA binding and transactivation activity, thus affecting Jun-mediated signal transduction and gene expression [51,52,53]. In a study on Alzheimer’s disease (AD), RPL10 was shown to be involved in cell death regulation, taking part in the signaling cascade induced by Presenilin 1 (PS1), which is a causative gene for an autosomal dominant familial AD. In this study, RPL10 was shown to bind to and colocalize with PS1 in the nuclear matrix in human brain neurons, as well as to bind to c-Jun, thus affecting transcription and apoptosis [49]. Oh and colleagues showed that human RPL10 interacts with c-Yes, belonging to a family of kinases that are involved in many intracellular functions, including cell stability, proliferation, migration, and differentiation. The described association of RPL10 results in c-Yes inhibition, thus affecting the downstream signal transduction [10]. In pancreatic tumors cells, it has been demonstrated that RPL10 specifically binds to p65 and IκB Kinase γ (IKKγ) Nuclear Factor-κB (NF-κB) family members, thus modulating the expression of key regulators in the NF-κB- pathway by virtue of an autogenous role in the regulation of their translation [54]. In the same context, RPL10 has been identified as a downstream effector of the NF-κB Inducing Kinase (NIK)-mediated antiviral signaling pathway; in vitro and in vivo studies in Arabidopsis Thaliana models showed that RPL10 is specifically phosphorylated by NIK, and that inactivation of the RPL10 gene enhances virus susceptibility [55]. In yeast, Ferreyra and Chiocchetti attributed to RPL10 extra-ribosomal functions in UV and oxidative stress response [28,56]. Chiocchetti and colleagues described an altered redox-sensitive protein signature in peripheral cells derived from patients with autism disorders, carrying a mutation in RPL10 [H213Q, see below]. They suggested that this differential pattern is compatible with cellular functions in the presence of physiological ROS levels, while in a context of high oxygen consumption and metabolism, such as in neural cells, this signature fails to balance the high oxidative stress, while impacting cellular metabolisms and, ultimately, synaptic functions [28]. In addition, the RPL10 homolog in tomato has been recently shown to suppress BAX-induced cell death in yeast through the inhibition of reactive oxygen species (ROS) generation [57].

Even though the extra-ribosomal functions of RPL10 have not been univocally linked to any specific trigger, they seem to be all associated to proliferation or stress conditions that impose a reprogramming of cellular metabolism to respond to an increased biosynthetic and/or bioenergetic demand. However, these situations are often not sharply distinguishable from those impacting on ribosomal function and translational activity. Therefore, further studies are needed to elucidate the mechanisms underlying the activation and regulation of RPL10 extra-ribosomal functions, and importantly if and how they are interconnected with ribosomal functions.

## 5. RPL10: A Player in Inherited Ribosomopathies

Ribosomopathies are a group of inherited developmental disorders stemming from aberrant ribosome production, in terms of either number or structure (reviewed in [8,58]). This group includes a small number of diseases [Diamond Blackfan Anemia (DBA), Schwachman-Diamond Syndrome, Treacher-Collins Syndrome, X-linked Dyskeratosis Congenita, Cartilage-Hair Hypoplasia, Bowen–Conradi Syndrome, and others] caused by mutations in genes encoding for ribosomal proteins or ribosome biogenesis factors, and they are characterized by very heterogeneous phenotypes, ranging from hematological disorders to morphological abnormalities (reviewed in [59]). Despite the wide range of clinical manifestations, a common feature of several ribosomopathies is an increased cancer predisposition [60].

The cause-effect link between mutations and clinical outcome is still a knot that needs to be unraveled, and this applies to all ribosomopathies described thus far. One of the most puzzling aspects in the pathogenesis of these diseases is that ubiquitous ribosomal alterations only have an impact on the correct development or function of some tissues or organs. These tissues are generally (but not consistently) highly proliferating tissues, such as bone marrow or epithelia (reviewed in [8]).

Three possible, and not necessarily mutually exclusive, mechanisms have been suggested as causing a transition from ribosome defect to disease. The first theory postulates that the correct translation of specific mRNA subsets is impinged by a decreased cellular concentration of mature ribosomes [58]. This possibility has been intensely explored in the context of DBA, where mutations in RP-encoding genes frequently generate RP haploinsufficiency, possibly reducing the availability of mature, functionally competent, ribosomes [61]. This reduction, albeit not impacting on global protein synthesis, would specifically decrease the translation of subsets of mRNAs, even though it is not clear, yet, on which bases [58,62]. The second theory is centered on the activation of the ribosomal stress response. This is triggered by an unbalance in the synthesis of ribosomal components, that, by altering the equilibrium of ribosome assembly, in turn determines an increase in the availability of free RPL5-RPL11-5S rRNA complexes. These complexes, which do not become incorporated in mature ribosomes, mediate MDM2 degradation, thus leading to p53 stabilization and activation (for an overview of this topic the reader is referred to [63]). The third theory suggests a central role for ribosome specialization in the molecular pathogenesis of ribosomopathies (reviewed in [64,65]). Specialized ribosomes are ribosomes with a unique composition (in terms of rRNAs and/or RPs and/or of their modifications) and translational activity. It has been posited that in physiological conditions, different sub-populations of ribosomes, characterized by diverse compositions and activities, may co-exist in each cell, and that they might modulate the translation of specific transcripts, thereby adding a new level of gene expression control [64]. In this context, ribosome specialization might play an important role in pathologic processes as well: for instance, the presence of ribosomes equipped with mutant ribosomal proteins could contribute to modifying the translation of specific mRNAs, by creating an imbalance in protein synthesis and, ultimately, leading to disease. As a paradigm of this concept, in the context of the above-mentioned DBA, it has been demonstrated that ribosomes incorporating a mutant variant of RPL9 have an intrinsic defect at the basis of the pathogenesis of the disease [66].

This may be the case also for a few inherited diseases or disorders, described in the past 15 years, all of which are attributed to mutations in RPL10. In all these instances of mutant RPL10 inheritance, transmission is X-linked with a recessive pattern. The first-ever report of a disorder associated with RPL10 mutations dates back to 2006. At that time, Dr. Klauck and colleagues described two subjects affected by autism, with no mutations in the genes formerly considered to be involved in the onset of this neurodevelopmental syndrome [13]. Indeed, these subjects had different mutations in the RPL10 gene (L206M and H213Q), which appear to involve conserved residues mapping quite close to each other in the protein [13,67] (Figure 2 and Table 1). Functional analyses of the RPL10 mutations identified in the autistic patients suggested that the two mutant forms of RPL10 may be functional hypomorphs that decrease the translational capacity of ribosomes, ultimately impacting on the synthesis of the cell surface components needed for synaptic plasticity and correct neurodevelopment [13].

Another amino acid substitution in the RPL10 sequence was subsequently reported to impact the development of the central nervous system and of the head [48,68]. RPL10 K78E mutation (Figure 2 and Table 1) described in an individual affected by mental retardation, seizures, and microcephaly was studied in a zebrafish model. These studies indicated that K78E substitution disrupted the interaction with 28S rRNA, altering translational efficiency, thus decreasing translation rate and increasing apoptosis in specific areas of the brain. In the same α-helix, A64V substitution (Figure 2 and Table 1) was reported in two individuals affected by intellectual disability, cerebellar hypoplasia, and skeletal abnormalities [19]. The mutation was proposed, based on its position within the functional domain between amino acids 59–94 of the protein (see above), to affect ribosome movement/rotation. As mentioned earlier, an open question applying to all ribosomopathies is: Why do mutations in ubiquitously expressed proteins only cause tissue-specific defects? For RPL10-related ribosomopathies, a possible explanation indicated by the existing literature [13,48] is that RPL10 basal expression is enriched in some specific areas of the brain compared to other tissues. There is some evidence suggesting that in those areas, there is a need for increased ribosome concentration to satisfy the request for prompt protein synthesis [13]. However, further studies are needed to clarify this point definitively and also to explore the possibility that in this context, RPL10 may be required also independently of its known ribosome-related function.

## 6. RPL10: A Player in Cancer

In recent years, it has become clear that somatic mutations (including point mutations and copy number alterations) in genes encoding RPs are a common feature of many cancer types (including solid and hematopoietic malignancies), suggesting their importance in oncogenesis [70,71,72,73]. Moreover, the expression deregulation of some RPs is detectable in a variety of tumors and has been suggested as being predictive of outcome [74]. In this context, RPs may play a role in driving the oncogenic process, acting as oncogenes (in the case of over-expression) or conversely as tumor-suppressors (in the case of haploinsufficiency).

Although RPL10 contribution to cancer was initially proposed almost 30 years ago [75], this issue has been intensely studied only in the past decade, after mutations in its sequence were described in the context of pediatric T-cell acute lymphoblastic leukemia (T-ALL) [76]. Here, some mutations involving two different amino acids (R98S, R98C, and Q123P) were detected and recognized for their active role in driving the oncogenic process. R98 is actually a mutational hotspot, since mutations in RPL10 involve this residue in over 90% of the RPL10 mutations (and about 8% of infant T-ALL cases). R98 and Q123 (H123 in yeast) form the basis of an essential flexible and dynamic loop in RPL10 that closely approaches the peptidyl-transferase center in the catalytic core in ribosomes, and which is involved in ribosome rotation during translation [77] (Figure 2 and Table 1). R and Q are both polar amino acids, and R has highly basic properties; their replacement with neutral/nonpolar amino acids may disrupt their interactions, possibly impacting the flexibility of the loop, on subunits assembly, and therefore on ribosome structure and function. R98S RPL10 mutation has been extensively studied for its impact on ribosome and for its role in cancer onset. R98S has been shown to indirectly affect the release of Nmd3 on the subunit interface [37]. In turn, Nmd3 retention inhibits Sdo1 binding and Tif6 release [26], thus impairing ribosome biogenesis and subunit assembly [24,37]. In addition, R98S has been shown to impinge on translational fidelity and to ultimately decrease proliferation in yeast and mammal (including human) cells, while increasing their resistance to otherwise lethal stimuli, such as oxidative stress and overgrowth [24,53,76]. Translational fidelity defects may alter the balance of protein synthesis, shifting it to a more pro-oncogenic phenotype. Indeed, a proteomics approach on a mouse-derived model of R98S mutation showed that the mutation triggers a change in the translatome, accounting for 4% of all translated proteins and involving numerous cancer-associated pathways such as metabolic reprogramming, reduced DNA repair, and hyper-activation of the Janus Kinase/Signal Transducer and Activator of Transcription (JAK/STAT) signaling cascade, which is a well-known player in T-ALL [78]. In addition, among the proteins whose synthesis was (up- or down-) regulated by R98S, there were numerous transcription factors, meaning that the mutation can also have an impact on gene expression upstream, by indirectly controlling transcription [79]. Furthermore, it has been demonstrated that R98S RPL10 is a potent driver of internal ribosome entry site (IRES)-mediated (5′ Cap-independent) Bcl2 translation, with a mechanism of action that may possibly explain the increased resistance to pro-apoptotic stimuli [53].

Even though most of the scientific literature about RPL10 alterations in cancer is related to T-ALL, this RP has been linked to other hematological and solid tumors. As far as the first are concerned, three RPL10 mutations have been described with low frequency (2%) in multiple myeloma [80]. These mutations (I33V, I70M, and I70L) cluster in a mutational hotspot localizing to a portion of the protein different from the one described for T-ALL -associated mutations (Figure 2 and Table 1). Based on the position of the involved residues within the protein and the ribosome, it was suggested that these mutations might affect ligand (tRNA) binding rather than ribosome biogenesis. An impact on cellular growth, ribosome biogenesis, and translation was demonstrated only for the I70L substitution in yeast models. However, based on the clustering and high conservation of I33 and I60 residues throughout the species, and on the position of these residues within the catalytic core of ribosomes, it is conceivable that all these RPL10 variants can significantly contribute to tumorigenesis in the context of multiple myeloma, by impacting on the translation of specific transcripts [80].

In the context of solid tumors, RPL10’s contribution in driving oncogenic processes has been suggested in pancreatic [50,54], prostatic [81], ovarian [12,82], and theratoid/rhabdoid tumors [83]. Even though these studies have not explored whether or not RPL10 is involved by virtue of its ribosomal/translation-related functions, its (higher) protein expression in tumors was linked to an increased cell proliferation and invasion [12,54], as well as an increased resistance to oxidative stress [50]. Therefore, even in different tumor types, RPL10 alterations seem to have a common set of consequences on cell behavior and may possibly contribute to cancer onset.

## 7. Summary and Outlook

For decades, ever since their discovery in the 1950s, ribosomes have been seen as passive operators in the assembly line of proteins. However, in the late 1990s, the identification of the first ribosome-associated inherited diseases (ribosomopathies) started to spread a new vision of ribosomes as active regulators of gene expression. This new concept has become more and more popular in the scientific community, as evidence linking ribosome alterations to disease has continued to accumulate. In recent years, in this context, ribosomal proteins have been recognized as a new class of factors with an active role not only in ribosomopathies, but also in cancer onset and progression. Among ribosomal proteins, RPL10 has been extensively studied for its role in the onset of inherited and acquired diseases, as discussed above. A number of studies, mainly in yeast and humans, have shed light on the role of RPL10 in ribosome assembly and function, thereby providing a rationale for the detrimental effects of its alterations on disease. In cancer, one of these effects has been proven to be the resistance to oxidative stress, which threatens cancer cell survival. In this sense, RPL10 mutations seem to slow down the rush of cancer cells toward uncontrolled growth, metabolism, and accumulation of toxic products, which could become unbearable in the long run. In this sense, it is possible that RPL10 mutations may provide a protective effect for cancer cells. Yet, it is worth underscoring that the mutations found so far in hematologic malignancies and in other tumors seem to cluster at tumor-specific hotspots in the 3D conformation of the protein (see Figure 2). It could be suggested that these mutations are particularly advantageous and thus selected for. This selection could be based on the cellular context where they develop (e.g., T- vs. B- cells), where different sets of cellular mRNAs and/or of mRNA binding factors may be cell-type specific. However, many aspects of the pathogenetic effect of RPL10 alterations are yet to be discovered. In particular, a mechanistic insight into how inherited alterations of RPL10 may condition neurodevelopment is completely lacking.

Clarifying the mechanisms that link RPL10 to disease will pave the way to the development of drugs that can specifically target the disrupted pathways downstream of RPL10 alterations. In parallel, in the perspective of a precision medicine approach, the identification of (new) compounds directly targeting mutant RPL10-incorporating ribosomes, may offer the possibility of directly rescuing the defect at its origin. Actually, the use of drugs directly targeting the translational machinery is already a therapeutic option in some instances of acquired disease and of cancer (reviewed in [84]). The rational design of molecules that specifically bind to mutant RPL10, rescuing its defect, or that interact specifically with defective ribosomes, inhibiting their abnormal translational activity, may lead to the availability of new therapeutic options for those diseases primarily caused by RPL10 alterations.

## Figures and Tables

**Table 1:** RPL10 variants described in human pathology; rows with a yellow background are those containing information of RPL10 mutations in inherited diseases, rows in light blue are those related to cancer, the row in green is related to both. CDS, coding sequence; aa, amino acid.

CDS Position	aa Substitution	Pathology	Reference
c.191C > T	A64V	intellectual disability, cerebellar hypoplasia, skeletal abnormalities	[13]
c.232A > G	K78E	mental retardation, seizures and microcephaly, epilepsy	[37,56]
c.481G > A	G161S	syndromic intellectual disability	[69]
c.605G > A	S202N	intellectual disability	[9]
c.616C > A	L206M	autism	[9,55]
c.639C > G	H213Q	autism, pancreatic cancer	[9,20,40,44]
c.97A > G	I33V	multiple myeloma	[68]
c.197A > G	E66G	multiple myeloma	[68]
c.208A > C	I70L	multiple myeloma	[68]
c.210T > G	I70M	multiple myeloma	[68]
c.292C > A	R98S	infant T-ALL	[64]
c.292C > T	R98C	infant T-ALL	[64]
c.368A > C	Q123P	infant T-ALL	[64]
